# Follow-up study of unilateral renal function after nephrectomy assessed by glomerular filtration rate per functional renal volume

**DOI:** 10.1186/1477-7819-12-59

**Published:** 2014-03-19

**Authors:** Yukinari Hosokawa, Nobumichi Tanaka, Hisakazu Mibu, Satoshi Anai, Kazumasa Torimoto, Tatsuo Yoneda, Akihide Hirayama, Katsunori Yoshida, Yoshiki Hayashi, Yoshihiko Hirao, Kiyohide Fujimoto

**Affiliations:** 1 Department of Urology, Tane General Hospital, 1-12-21 Kujyominami Nishi-ku, Osaka, Osaka 550-0025, Japan; 2 Department of Urology, Nara Medical University, 840 Shijo-cho, Kashihara, Nara 634-8522, Japan

**Keywords:** Functional renal parenchymal volume, eGFR, Proteinuria, Renal surgery

## Abstract

**Background:**

To evaluate the clinical usefulness of estimated glomerular filtration rate (eGFR) divided by functional renal volume (FRV) measured by three-dimensional image reconstruction (eGFR/FRV) for the prediction of functional outcomes after nephrectomy.

**Methods:**

Eighty-three patients who underwent nephrectomy were enrolled. The FRV of each patient was measured before surgery. Preoperative medical information on proteinuria, blood pressure, blood glucose level, body mass index (BMI), hemoglobin level and serum cholesterol level were also obtained. We evaluated the relationships between eGFR/FRV and each of these parameters before surgery. We also assessed the potential relationship between eGFR/FRV and the 3-year postoperative eGFR. Stepwise multiple regression analyses were conducted to elucidate independent factors.

**Results:**

The median FRV and eGFR were 310.15 cm^3^ and 79.0 ml/min/1.73 m^2^ before surgery, respectively. The correlation between FRV and eGFR was statistically significant (r = 0.465, *P* < 0.001). The median eGFR/FRV was 0.24 ml/min/1.73 m^2^/cm^3^. Stepwise multiple regression analysis showed that the independent parameters (multiple correlation coefficient, r = 0.389, *P* = 0.031) associated with eGFR/FRV were proteinuria, BMI, age and hypertension. Proteinuria was statistically associated with eGFR/FRV, and the independent parameters (multiple correlation coefficient, r = 0.694, *P* < 0.001) associated with the 3-year postoperative eGFR were age, BMI and eGFR/FRV. The eGFR/FRV was statistically associated with the 3-year postoperative eGFR (r = 0.559, *P* < 0.001).

**Conclusion:**

The present results demonstrated that patients with proteinuria are expected to have a lower eGFR/FRV than those without proteinuria. The present study also supports the notion that eGFR/FRV is the primary determinant of the long-term functional outcome after nephrectomy. It should be taken into consideration that patients with a low eGFR/FRV may develop chronic kidney disease after nephrectomy.

## Background

Most patients who are newly diagnosed with renal cell carcinoma (RCC) are usually treated surgically. Recently, nephron sparing surgery (NSS) is considered to be as curative for early stage RCC as radical nephrectomy (RN) [1] and is becoming a standard therapy for small RCC. Several groups have reported that RN increases the risk for adverse renal and cardiovascular morbidity [2-4]. NSS also resulted in cancer-specific survival equivalent to RN [5]. However, RN remains the most common surgery for small renal tumors [6]. From the perspective of the prevention of postoperative renal insufficiency, the prediction of postoperative renal function is definitely an important issue in patients who undergo renal surgery. Accurate prediction of postoperative renal function should be considered before renal surgery is performed.

We have previously reported that postoperative renal function can accurately be predicted before surgery by using both the preoperative serum creatinine level (sCr) and the functional renal volume (FRV), which is estimated by three-dimensional reconstructed software and diagnostic images (computed tomography (CT) scan and magnetic resonance imaging (MRI)) [7]. This prediction was not only available for patients who underwent RN, but also for those who underwent NSS. Although FRV showed a significant correlation with sCr and creatinine clearance (CCr) [7], the absolute values of sCr and CCr varied between patients with similar FRV. This finding may be caused by the difference in patients’ age or gender, and comorbidities such as hypertension, cardiovascular disease, hyperlipidemia, diabetes mellitus, renal insufficiency, or other conditions.

The meaning of renal function per FRV in renal surgery is unknown. It would be informative and meaningful to clarify the clinical significance of renal function per FRV in association with perioperative and long-term management after renal surgery, the evaluation of renal function in healthy individuals, and the assessment of renal insufficiency. We conducted the present study to elucidate this issue.

## Methods

### Ethical approval

The institutional review board approved this study, and informed consent was obtained from all patients after explaining the aim and methods of this study.

### Patient selection

Eighty-three patients who consecutively underwent nephrectomy or donor nephrectomy, and for whom preoperative FRV was calculated using three-dimensional reconstruction software between 2006 and 2008 at Nara Medical University and its affiliated hospital were enrolled. These patients consisted of 44 men and 39 women. Their mean age was 60.1 years (median 60.0 years, range 26 to 89 years). Of all of these patients, 45 underwent RN for RCC, 9 underwent nephroureterectomy for upper urinary tract carcinoma, and 29 underwent donor nephrectomy.

### Preoperative and postoperative renal function assessment

To calculate FRV, enhanced CT scans and MRI images were input into the computer program. We used a dynamic image of the arterial phase to determine the functional renal parenchyma. For each diagnostic image, the renal parenchyma was traced by erasing the neighboring organs, fat tissue, muscles, and collecting system which are not of interest using the computer mouse. The traced outlines of the renal parenchyma in all the images were combined to automatically reconstruct a three-dimensional image. The FRV was estimated by integral calculation software (MU1128, developed by Y Yamazaki Y, and commercially unavailable). The estimated glomerular filtration rate (eGFR) was adopted as the renal function. The eGFR was calculated from sCr and age using the following equation: eGFR (ml/min/1.73 m^2^) = 194 × sCr^ -1.094^ × age ^-0.287^ (× 0.739, for females). This equation was revised for the Japanese population from the original equation of the Modification of Diet in Renal Disease (MDRD) study by the Japanese Society of Nephrology [8].

We evaluated the correlation between preoperative eGFR and FRV in all the patients. The renal function per FRV was calculated as eGFR divided by FRV (eGFR/FRV). To elucidate the parameters that correlated significantly with eGFR/FRV, the following parameters were tested: age, gender, anemia, body mass index (BMI), hypertension, proteinuria, hyperlipidemia, and diabetes mellitus. The cut-off value of hyperlipidemia was either a serum cholesterol level of 220 mg/dl or greater or a serum triglyceride level of 150 mg/dl or greater [9]. If patients were receiving medication for hyperlipidemia, they were categorized as hyperlipidemia regardless of the cut-off value. Patients with a fasting blood sugar level of 126 mg/dl or greater, or an HbA1c level of 6.5% or greater, were categorized as having diabetes mellitus according to the definition by the Japan Diabetes Society [10]. Patients who were receiving medication for diabetes mellitus were also defined as having diabetes mellitus. The cut-off value of proteinuria was defined as a positive result on the paper strip test. Patients who showed a systolic blood pressure of 140 mmHg or greater or a diastolic blood pressure of 90 mmHg or greater were categorized as having hypertension. This stratification was defined as first-degree hypertension by the Japanese Society of Hypertension [11]. Patients who were receiving medication for hypertension were also defined as having hypertension. Patients were followed for 3 years or longer.

### Statistical analysis

Spearman’s rank correlation test was used to assess the correlation between eGFR and FRV. The Mann–Whitney U test was used to compare eGFR/FRV between patients with and without preoperative comorbidities. Stepwise multiple regression analysis was used to elucidate the significant parameters correlating to eGFR/FRV (step-up method: Fin = Fout = 2.0). Stepwise multiple regression analysis was also used to investigate parameters independently determining the 3-year postoperative eGFR. All statistical analyses were carried out using SPSS 17.0 J (SPSS Inc., Chicago, IL, USA). *P* < 0.05 was regarded as statistically significant in all statistical tests.

## Results

**Figure 1:**
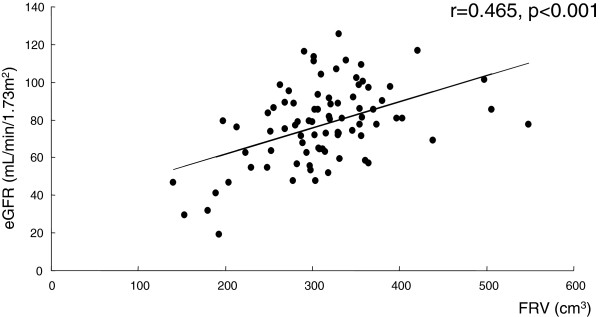
**Significant correlation between estimated glomerular filtration rate (eGFR) and funtional renal volume (FRV) estimated by three-dimensional image reconstruction (r = 0.465, *P*** **< 0.001).** Note that the distribution of eGFR varies between individuals with similar FRV.

Table 1 shows the patients’ characteristics. The median values of the preoperative FRV and the eGFR were 310.15 cm^3^ and 79.0 ml/min/1.73 m^2^, respectively. Regarding concurrent diseases, 61.4% of the patients had hypertension, the most common concurrent disease, and 15.7% of the patients had proteinuria, the least frequent concurrent disease. Although there was a significant correlation between FRV and eGFR by Spearman’s rank correlation test (r = 0.465, *P* < 0.001) (Figure 1), the correlation coefficient was moderate. Indeed, the distribution of eGFR varied between individuals. There was a difference in eGFR of approximately 80 ml/min/1.73 m^2^ in patients with similar FRV.

**Table 1:** Characteristics of 83 patients with functional renal volume measurements

**Parameter**	
Men age (years)	60.1 (range 26–89; median 60.0)
Gender (male/female)	44/39
Mean BMI (kg/m^2^)	23.2 ± 4.1 (range 17.2-45.0; median 22.8)
HT (yes/no)	51/32 (under treatment n = 25)
Diabetes (yes/no)	15/68 (under treatment n = 6)
Hyperlipidemia (yes/no)	39/44 (under treatment n = 7)
Anemia (yes/no)	18/65
Proteinuria (yes/no)	13/70
eGFR (ml/min/1.73 m^2^)	77.5 ± 21.0 (range 19.0-125.7; median 79.0)
eGFR (ml/min/1.73 m^2^)/FRV (cm^3^)	0.25 ± 0.06 (range 0.10-0.40; median 0.24)

BMI, body mass index; eGFR, estimated glomerular filtration rate; FRV, functional renal volume; HT, hypertension.

The mean eGFR/FRV was 0.25 ml/min per 1.73 m^2^/cm^3^ (SD 0.06, median 0.24). Each value of eGFR/FRV stratified by clinical parameters was investigated. There were significant differences in eGFR/FRV depending on gender (male = 0.22 ml/min per 1.73 m^2^/cm^3^ versus female = 0.27 ml/min per 1.73 m^2^/cm^3^, *P* = 0.012), hypertension (yes = 0.23 ml/min per 1.73 m^2^/cm^3^ versus no = 0.27 ml/min per 1.73 m^2^/cm^3^, *P* = 0.033), proteinuria (yes = 0.20 ml/min per 1.73 m^2^/cm^3^ versus no = 0.26 ml/min per 1.73 m^2^/cm^3^, *P* < 0.001) and BMI (≥22 kg/m^2^ = 0.22 ml/min per 1.73 m^2^/cm^3^ versus <22 kg/m^2^ = 0.28 ml/min per 1.73 m^2^/cm^3^, *P* < 0.001), while there were no significant differences between diabetes, hyperlipidemia and anemia. The independent parameters associated with eGFR/FRV were proteinuria, BMI, age, and hypertension (multiple correlation coefficient, r = 0.389, *P* = 0.031). Of these four parameters, proteinuria is the only significant parameter in the multiple regression analysis (standardized partial regression coefficient −0.253, *P* = 0.042).

At the last follow up, 10 patients had died of cancer and 17 patients had been lost to follow-up. The 3-year postoperative median eGFR was 50.1 ml/min/1.73 m^2^. The median follow-up period was 36.0 months (mean 27.1 months). There was a significant decrease in eGFR at 1 month after nephrectomy, but no further significant changes thereafter. There was no significant relationship between the reduction rate of the 1-month postoperative eGFR and eGFR/FRV (r = −0.211, *P* = 0.119).

**Figure 2:**
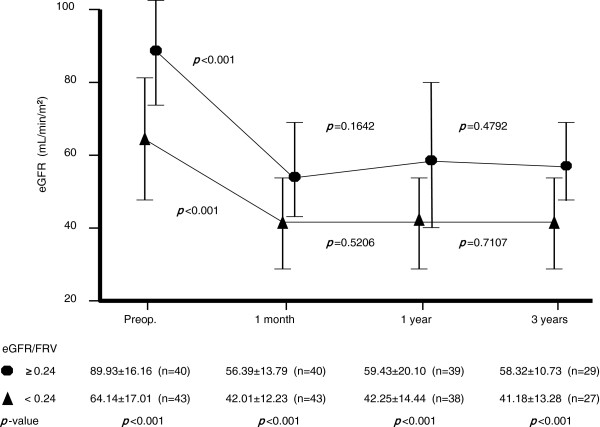
**Estimated glomerular filtration rate (eGFR) change of the contralateral kidney after nephrectomy in the two groups divided by an eGFR/FRV cutoff at 0.24.** Patients with an eGFR/FRV of ≥0.24 had significantly better renal function than those with an eGFR/FRV of <0.24 at 3 years postoperatively. FRV, functional real volume.

The patient characteristics divided by an eGFR/FRV cutoff at 0.24 are presented in Table 2. This cutoff value is defined as the point with the most significant (Mann–Whitney test) split between preoperative eGFR/FRV and the 3-year postoperative eGFR. Comparison of the reduction rate between the preoperative eGFR and the 1-month postoperative eGFR did not show a significant difference between the eGFR/FRV ≥0.24 and the eGFR/FRV <0.24 groups (*P* = 0.4355). Compared to low eGFR/FRV (<0.24), high eGFR/FRV (≥0.24) were younger, had lower BMI and high eGFR. There were also significant differences in gender, diabetes and proteinuria, while there were no significant differences between hypertension, hyperlipidemia and anemia. Patients under 60 years, those without hypertension or hyperlipidemia and those with an eGFR/FRV of more than 0.24 had significantly superior preoperative renal function. Patients with an eGFR/FRV of ≥0.24 retained significantly better renal function than those with an eGFR/FRV of <0.24 at 3 years postoperatively (Figure 2). Gender, BMI, diabetes mellitus, anemia and proteinuria did not have a significant influence. The independent parameters associated with 3-year postoperative eGFR are age, BMI and eGFR/FRV (multiple correlation coefficient, r = 0.694, *P* < 0.001). On multivariate analysis, age and eGFR/FRV were the significant parameters (Table 3). The preoperative eGFR/FRV significantly correlated with the 3-year postoperative eGFR (r = 0.559, *P* < 0.001).

**Table 2:** Characteristics of 83 patients divided by an eGFR/FRV cutoff at 0.24

**eGFR (ml/min/1.73 m**^**2**^**)/FRV (cm**^**3**^**)**	**<0.24 (n = 40)**	**≥0.24 (n = 43)**	***P *** **value**
Age (years)	64.7 ± 9.6	55.8 ± 14.3	0.002
Gender (male/female)	26/14	18/25	0.035
BMI (kg/m^2^)	24.3 ± 4.8	22.2 ± 3.1	0.027
HT (yes/no)	28/12	23/20	0.123
Diabetes (yes/no)	11/29	4/39	0.031
Hyperlipidemia (yes/no)	21/19	18/25	0.332
Anemia (yes/no)	10/30	8/35	0.480
Proteinuria (yes/no)	13/27	0/43	<0.001
eGFR (ml/min/1.73 m^2^)	64.1 ± 17.0	89.9 ± 16.2	<0.001
Donor nephrectomy (yes/no)	10/30	19/24	0.067

BMI, body mass index; eGFR, estimated glomerular filtration rate; FRV, functional renal volume; HT, hypertension.

**Table 3:** Multiple regression analysis of factors determining 3-year postoperative eGFR

**Parameters**	**Standardized partial regression coefficient**	***t *** **value**	***P *** **value**
Age	−0.376	−3.590	0.001
Gender (female versus male)	*	*	0.787
BMI (<22 kg/m^2^ versus ≥22 kg/m^2^)	0.165	1.622	0.111
HT (no versus yes)	*	*	0.891
Diabetes (no versus yes)	*	*	0.797
Hyperlipidemia (no versus yes)	*	*	0.967
Anemia (no versus yes)	*	*	0.923
Proteinura (no versus yes)	*	*	0.796
eGFR/FRV	0.489	4.588	<0.001

*Factors that were not selected by means of stepwise multiple regression analysis.

Multiple correlation coefficient, r = 0.694, *P* < 0.001. BMI, body mass index; eGFR, estimated glomerular filtration rate; FRV, functional renal volume; HT, hypertension.

## Discussion

The deterioration of renal function after renal surgery (for example, nephrectomy, NSS and nephrouretectomy) is a major concern. Recent observations suggest that RN significantly increases the risk of *de novo* chronic renal insufficiency compared with PN [2,3]. It was also noted that a decreased eGFR is associated with an increased risk of death, cardiovascular events and hospitalization [2-4]. However, RN is commonly performed for RCC in the USA [6]. The prediction of postoperative renal function is a meaningful approach to reduce the risk of postoperative renal insufficiency. Many reports have discussed the predictors of residual renal function after RN and NSS [3,12-14].

In the USA, it is common to use the MDRD equation to calculate eGFR [15]. Accurate calculation of eGFR for each ethnic group requires modification of the equation. Therefore, the equation was revised for the Japanese population by using age, gender, and serum creatinine and the original equation of the MDRD Study by the Japanese Society of Nephrology [8]. In the present study, we used this equation to calculate eGFR. We have already reported that FRV can be calculated by diagnostic images (CT scan and MRI) and three-dimensional reconstruction software. We have also elucidated that the postoperative renal function and FRV can be predicted preoperatively by using preoperative renal function and FRV [7]. Gong and colleagues reported that CT-estimated FRV strongly correlated positively with renal function and correlated inversely with age [16]. However, no one has studied eGFR/FRV. Our results indicate that the distribution of eGFR varied between individuals with similar FRV, but there was a moderate correlation between eGFR and FRV. The multiple regression analysis revealed that proteinuria is the only parameter that influences the eGFR/FRV. This result may reveal that glomerular function influences eGFR/FRV. It is well known that proteinuria is a risk factor for renal insufficiency. Imai and colleagues reported that patients with positive proteinuria showed a significant decrease in eGFR of 5 ml/min/1.73 m^2^ in all generations between 40 and 80 years based on the 2005 Japanese annual health check program [17]. They concluded that proteinuria is strongly associated with renal insufficiency. They also reported that the decreasing rate of renal function in patients with positive proteinuria is around twice as high as that in patients without proteinuria in a longitudinal study using the Japanese annual health check program [18]. James and colleagues also reported that the risk factor of acute renal failure is strongly associated with eGFR and proteinuria in a cohort study in approximately 920,000 adults residing in Alberta, Canada [19]. Taken together, it seems that positive proteinuria is a risk factor for renal insufficiency after nephrectomy, as well as a risk factor for mortality in the normal population.

We also attempted simultaneous analysis of changes in eGFR after nephrectomy. In our series, eGFR/FRV was an independent predictor of the 3-year postoperative renal function and showed significantly correlation with the GFR at 3 years after nephrectomy (*P* < 0.001). Lindeman and Goldman reported that the age-related decrease in GFR in individuals with no specific renal disease was attributable to an involutional process leading to glomerular atrophy [20]. The differences in the eGFR/FRV in individuals could reflect glomerular function in individuals. Moreover, preoperative eGFR/FRV influenced postoperative renal function. This present study was focused on eGFR/FRV. However, both eGFR and FRV *per se* are also reported as a significant predictor for eGFR change of the contralateral kidney after nephrectomy [21]. We have also reported this issue [7]. Under these investigations, the relationship between FRV and renal function might be interesting. In other words, patients with the same renal function show different FRV. Consequently, we had the idea of using renal function per unit volume (eGFR/FRV).

There are several limitations to the present study. Firstly, the number of patients was small and the observation period was short. Moreover, 10 patients had died of cancer and 17 patients had been lost to the last follow-up. We must take into consideration the influence of these patients. Secondly, the definition of hypertension included patients who were already being treated with antihypertensive medication. It is controversial to combine patients who are treated with such medications as angiotensin II receptor blockers and untreated patients in the same cohort [22]. Thirdly, it is more complicated to handle patients with diabetes mellitus than normal patients, because diabetic patients in their 40s showed higher eGFR than those with a normal blood glucose level due to hyperfiltration [17]. Finally, our series included RCC, upper urinary tract carcinoma, and living kidney donors. It is generally noted that patients with RCC already had a decreased eGFR [23,24]. Whether there was hydronephrosis or not before surgery may influence the postoperative renal function.

Our present study and our previous report [7] reveal that there was a significant correlation between FRV and eGFR. Proteinuria was an independent factor that influenced the eGFR/FRV. Patients who undergo nephrectomy with an eGFR/FRV of <0.24 should be closely observed for the development of renal insufficiency.

## Conclusions

It is obvious that preoperative FRV and renal function play important factors in postoperative renal function. Further studies on the clinical usefulness of the eGFR/FRV measurement are necessary to elucidate the significant risk factors for the development of renal insufficiency after nephrectomy. This will allow us to identify those who should be on a strict follow-up schedule and who should receive daily life guidance to avoid renal insufficiency after nephrectomy. The scheduled monitoring of renal function and integration of the feedback analysis is expected to serve as a predictive tool for the development of medium- and long-term renal function.

## Abbreviations

BMI: body mass index; CCr: creatinine clearance; CT: computed tomography; eGFR: estimated glomerular filtration rate; FRV: functional renal volume; MDRD: Modification of Diet in Renal Disease; MRI: magnetic resonance imaging; NSS: nephron sparing surgery; RCC: renal cell carcinoma; RN: radical nephrectomy; sCr: serum creatinine level.

## Competing interests

The authors declare that they have no competing interests.

## Authors’ contributions

NT, SA, YH (Hirao Y) and KF designed the research; YH (Hosokawa Y), and HM conducted the research; NT and MH provided the radiological database; YH (Hosokawa Y), NT, HM, SA, KT, TY, AH, KY and YH (Hayashi Y) analyzed the data; YH (Hosokawa Y), NT and KF wrote the paper; KF had the primary responsibility for the final content. All authors read and approved the final version.
